# Youth victim perspective: optimizing presentation of patient-reported outcomes in a violence intervention program

**DOI:** 10.1186/s40621-023-00451-8

**Published:** 2023-07-25

**Authors:** Ashley Hollo, Mark Nimmer, Brooke Cheaton, Marlene Melzer-Lange, Michael Levas

**Affiliations:** 1 University of Colorado, Aurora, CO USA; 2 Department of Pediatrics, Medical College of Wisconsin, Milwaukee, WI USA; 3 Project Ujima, Milwaukee, WI USA; 4 Section of Emergency Medicine, Medical College of Wisconin, Milwaukee, WI USA

**Keywords:** Patient-reported outcome measures, Adolescent, Quality of life, Crime victims, Violence

## Abstract

**Introduction:**

The health, well-being and psychological development of children in urban areas is threatened by exposure to interpersonal violence. Violence intervention programs, such as Project Ujima, provide children with comprehensive treatment following exposure to violence. Services focus on the interruption of the violence cycle, mental health, and developing resiliency. The collection of patient-reported outcomes (PROs) from youth victims of violence informs community-based, programmatic, and individual participant interventions. Although the collection of PROs throughout treatment has been demonstrated to be feasible, youth and crime victim specialist preferences for data presentation is unknown. We sought to determine patient and crime victim specialist preferences regarding which PROs are of interest and how best to visually display them for optimal engagement.

**Results:**

Fifteen youth and nine crime victim specialists consented to participate. Both preferred visuals with the highest level of color-shading and descriptions. The domains with the highest level of interest among both youth and case workers were social, anger, emotional, school, physical, peer relations, and psychosocial well-being. Youth and crime victim specialists expressed low interest in positive affect, meaning/purpose, physical stress experience, and depression domains. Youth wanted to see their scores compared to others in the program, while crime victim specialists did not think such comparisons would be beneficial. In contrast to youth, crime victim specialists believed youth should see their physical functioning and PTSD scores.

**Conclusion:**

Youth participants and their crime victim specialists in a violence intervention program desired to see their PROs in a graphical form and agreed on their preference for many of the domains except for PTSD and physical functioning. Both groups preferred visuals with the highest level of shading and descriptions. Further investigation is needed to determine how to implement PRO visuals with the desired domains into regular violence intervention programming.

**Methods:**

Participants in Project Ujima’s 8-week summer camp, ages 7–18 years, who were either a victim of violent injury, a direct relative of a violent injury victim, or a homicide survivor were recruited for this qualitative study. Crime victim specialists, who work directly with these youth throughout the year, were also recruited to participate. We conducted structured interviews to determine which parameters and visual formats were of highest interest and best understood by youth participants and crime victim specialists.

## Introduction

According to the Centers for Disease Control and Prevention, gun violence is now the leading cause of death for American children (Matt McGough and Panchal 2022). Interpersonal violence ranks among the top five leading causes of youth seeking medical attention and is associated with decreased five year survival in victims (Cheng et al. 2003; Sims et al. 1989). Youth directly exposed to such violence are at risk for experiencing emotional and behavior problems, diminished school connectedness/participation, and repeat victimization or becoming a perpetrator in the future(Johnston et al. 2002; Zun et al. 2004; Cheng et al. 2008a, 2008b).

Project Ujima is a violence intervention program established in Milwaukee in 1995 in response to the growing violence epidemic. The program responds to the identified physical and psychosocial needs of youth and families affected by interpersonal violence and targets 350 youth annually who present with firearm or other assault injuries to the Children’s Wisconsin Emergency Department/Trauma Center, a level one trauma, tertiary care center. Crime victim specialists with Project Ujima respond to the hospital when a child is injured following an act of community or interpersonal violence. They work alongside youth and families for a period of 12–15 months to support their healing journey by addressing their basic needs and other areas of need related to social determinants of health.

Project Ujima offers a unique opportunity to improve the lives of children impacted by violence in Milwaukee by offering both individual and group wellness, youth development, and mental health activities.

**Fig. 1:**
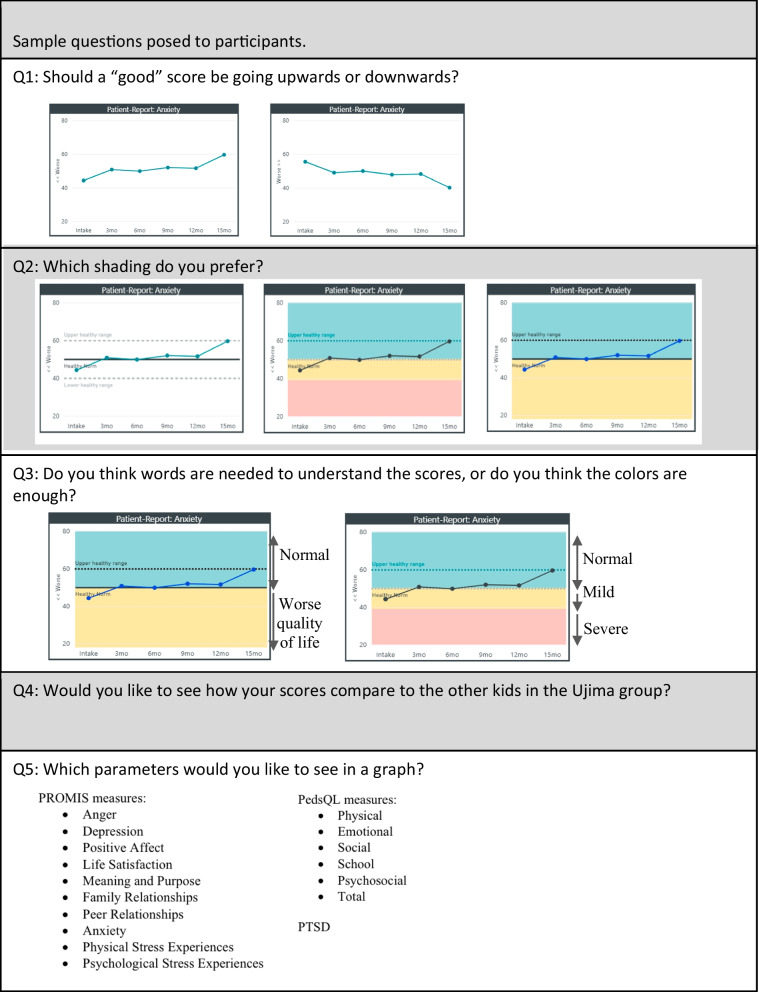
Sample questions posed to participants

Project Ujima collects data from youth participants through surveys taken every three months. The responses are quantified through the National Institute of Health’s Patient-Reported Outcomes Measurement Information System (PROMIS) (Cella et al. 2007). This allows a score, also known as a patient-reported outcome measure (PRO), to be calculated for each child (Fig. 1. Question 5). These patient-reported outcome (PRO) measures serve as outcome indicators and inform program leaders about youth responsiveness to programming. Project Ujima does not currently share this data with youth or family.

In one previous study of PRO visualization in pediatric oncology patients and their families, visuals with shading and written descriptions were preferred (Dobrozsi and Panepinto 2017). Limited research has been conducted to determine the most effective way to visually display PROs for pediatric patients who have been impacted by interpersonal violence. It is also unknown what specific measures parents, and their families wish to see throughout their violence intervention programming. This was an exploratory, qualitative study to determine which scored categories of PRO measures the youth were interested in seeing on a regular basis and how that compared to the interests of their crime victim specialists. The secondary purpose was to determine how to best graphically present scores to the youth in a way that was easy for them to interpret.

## Results

### Visual preference (Figs. 1 and 2)

**Fig. 2:**
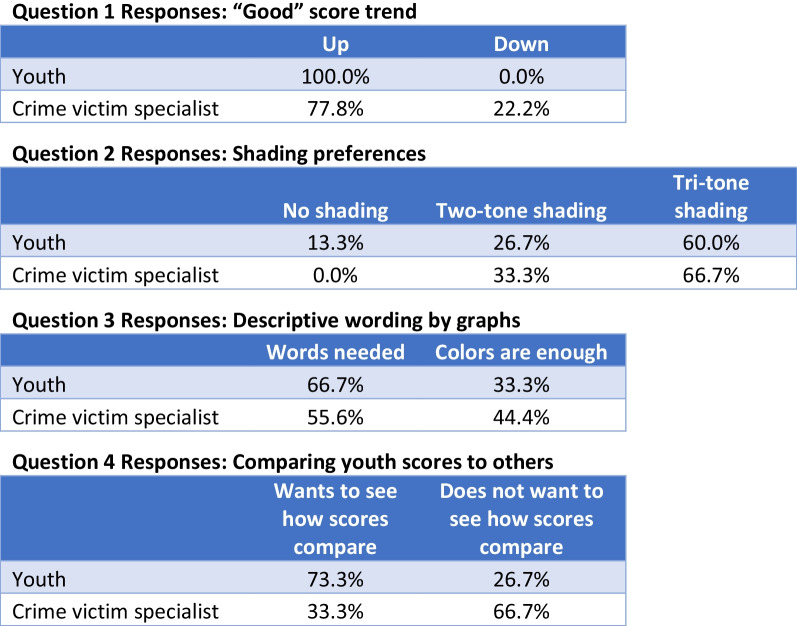
Sample questions and associated responses

15 youth and 9 crime victim specialists were interviewed. All Project Ujima youth and a majority of crime victim specialists preferred visuals in which a “good” score trended upwards. Most of the youth and crime victim specialists also chose the tri-tone “stoplight” color shading as a means of interpreting the scores. Both groups stated that words were needed adjacent to the colored shading for better understanding of the scores. After shading was determined, participants were asked about what type of labels they thought were helpful for interpreting scores. The mock visuals displayed the terms “normal,” “mild impairment,” and “severe impairment” next to each shaded region. These terms were deemed inappropriate by many crime victim specialists who stated that words like “normal” and “impairment” may be stigmatizing and negatively impact a child’s mental health. Crime victim specialists felt that the term “worse quality of life” was a better description for the yellow or red shading as it did not have as negative a connotation as “impairment.” Three children who chose to comment on the verbiage stated they did not understand the term “impairment.”

The only visual outcome on which youth and crime victim specialists’ preferences diverged was displaying individual scores with their peer average. The majority of youth preferred to see how their scores compared to others in the program, while most crime victim specialists did not think youth should see how their scores compare to their peers.

**Fig. 3:**
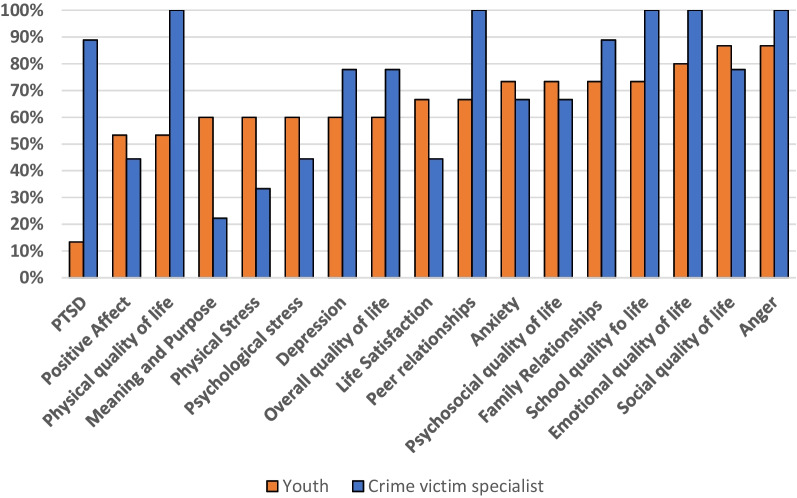
Question 5 responses: measures preferred to be seen in a graphical format

**Fig. 4:**
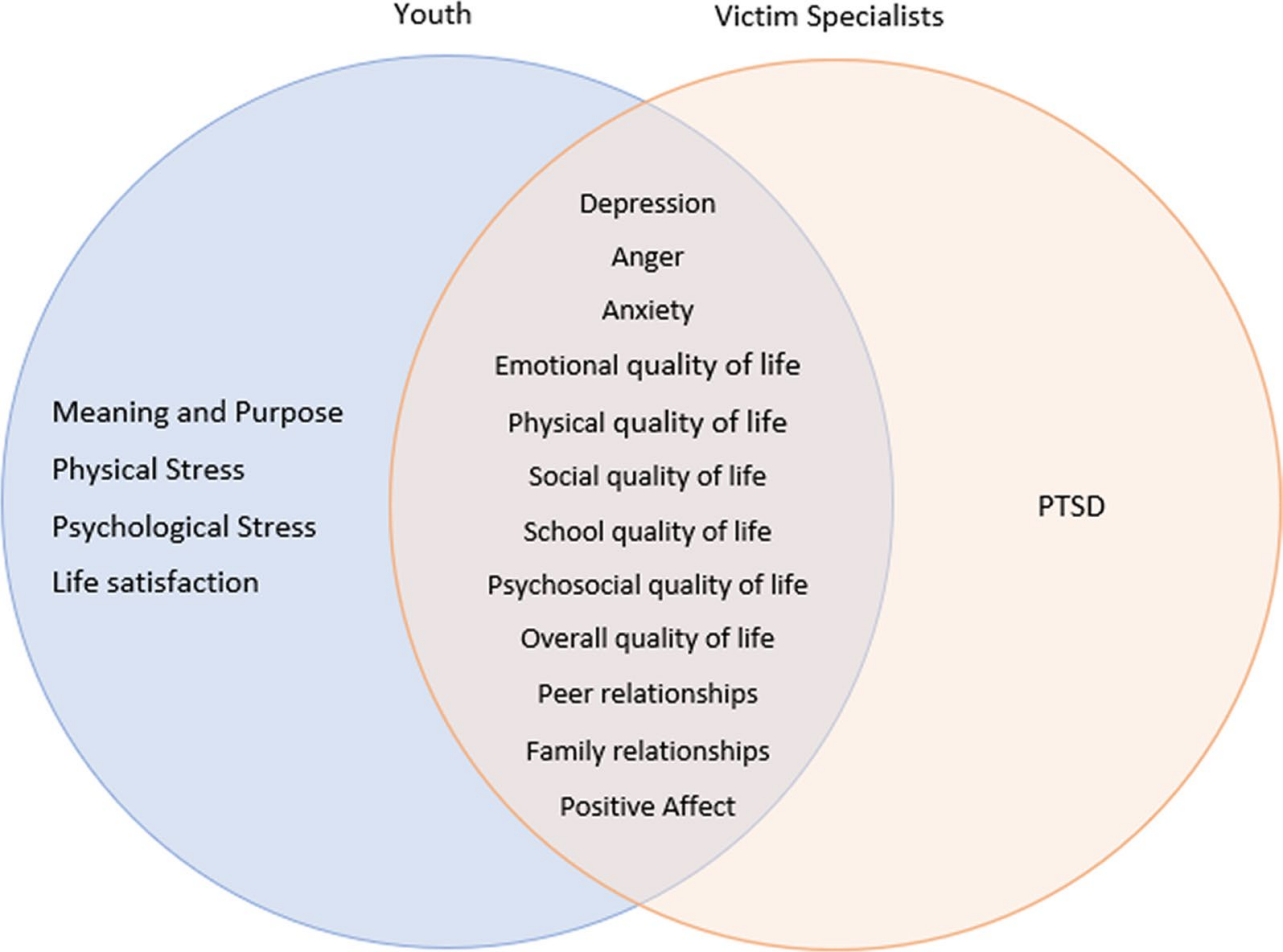
Overlapping and divergent preferences between youth and victim specialists

Crime victim specialists unanimously agreed that physical, peer relations, school, emotional, and anger outcomes be included in the visualization (Fig. 3). More than 80% of crime victim specialists also desired to see visuals displaying PTSD and family relationship scores. No PROs were unanimously preferred by youth; however, more than 70% of youth expressed a preference for viewing anxiety, psychosocial, family relationships, school, emotional, social, and anger scores visually. The largest difference between youth and crime victim specialist preferences was seen in PTSD scores; preferred by 13% of youth and 89% of providers. Figure 4 represents concordance between youth and victim specialist preferences. Responses were considered concordant if they were within 33.3% of each other.

## Discussion

This study affirms that most youth and crime victim specialists believed it would be advantageous for children to see a visualization of their PRO scores while participating in a violence intervention program. This provides another potential tool for participants of the program to get quantitative feedback on their progress following a violent victimization. Two youth did not believe seeing a graph of their progress would be useful, which may be attributable to the age of participants, with those who are at the younger range potentially being too young to properly interpret a graph. Regarding layout, a majority of those questioned also agreed that formatting the lines such that a “good” score was trending upwards was easier to understand. Two crime victim specialists disagreed with this, which may be because the mock graphs they saw displayed anxiety. It may have been counterintuitive for them to see a “good” anxiety score trending upwards, thus accounting for their response differing from the others. One crime victim specialist suggested that the x-axis of the graph be a date as opposed to the number of months from intake, so youth could have a better sense of their progress over time.

Additional questions addressed fine-tuning the graphical formatting to make sure the participants, aged 7–18, could easily interpret it with minimal assistance from an adult. Most youth participants chose the tri-tone colored shading. The two youth that did not were 13 and 14 years old and may have been old enough to interpret the graph without the assistance of colors. These findings were similar to those in a study of pediatric oncology patients, which concluded that patients and their families preferred graphs with the highest levels of visual and text interpretation of PROs (Dobrozsi and Panepinto 2017). The use of color-coding, and stop-light colored shading more specifically, is not new to the field of pediatrics. For example, color coding has been used in the pediatric Emergency Department to help patients accurately identify their level of pain (Bailey et al. 2007). Additionally, the “Traffic Light Diet,” which uses the stop-light colors to indicate which foods children should eat to be healthy, has been shown to facilitate greater weight loss than traditional weight loss methods in the treatment of pediatric obesity (Johnston and Steele 2007). Further research is needed to determine the most appropriate language to use other than the tri-tone shading on the graphs. Recognizing that health literacy varies greatly among children in the study age range of 7–18, further graphical formatting may require the language on each participant’s graphs to vary based on their age and reading level (Sanders et al. 2009).

The final question pertained to whether it would be beneficial for participants to compare their progress in the program to other youth. Most of the youth preferred to see their scores compared to others in the program, while most crime victim specialists did not believe these comparisons should be shared. It was suggested that sharing how youth scores compare to their peers may elicit feelings of shame or even create an unhealthy atmosphere of competition instead of that of personal improvement.

Compared to the other questions in the survey, there were more diverse answers regarding which scores the youth and crime victim specialists thought would be helpful to see (Fig. 4). All crime victim specialists believed that the youth would benefit from seeing visual displays of their physical, peer relations, school, emotional, and anger scores. Of these preferences chosen by crime victim specialists, youth agreed that school, anger, and emotional scores were important for them to see as well. Additional domains with the highest proportion of youth votes were for visuals displaying social, family relationships, psychosocial, and anxiety scores. Youth were less interested than the crime victim specialists in knowing about their physical wellbeing score. The crime victim specialists’ interest in having youth see their physical wellbeing scores could potentially stem from their first encounters being in a hospital setting due to the nature of the violence that the youth experienced, whereas these scores, potentially reflecting physical limitations, may be triggering for the youth. Another important difference between youth and crime victim specialist preferences was seen in the peer relationships and PTSD domains. It is possible youth do not fully understand how a prior victimization could affect current or future peer relationships, causing them to rank this metric as less important for their progress in the violence intervention program. It is also possible that the youth do not have the health literacy needed to fully comprehend what post-traumatic stress disorder is or how it may impact them as a victim of violence.

One limitation to this study was that all participants were the same race/ethnicity. Although this was representative of overall program enrollment, it prevented us from gaining insight into the preferences of children of other races/ethnicities or make comparisons between those children. Finally, there was potential for participant bias given that the youth were highly involved in the program. Next steps for this study involve using the graphs with the specifications determined by our results to show the youth their progress at regular intervals. Another logical next step would be to survey parents of participants, with the hope that in the future each youth could see the parameters that they have chosen (or their parents have chosen) to be important for their progress in the violence invention program. Finally, additional studies may allow us to determine if showing youth their PROs in a customized visual format throughout their time in the program enhances their ability to recover from violence.

## Conclusions

This study allowed us to take a closer look at youth and crime victim specialist preferences for optimal visualization of PROs in a violence intervention program. Youth and crime victim specialists preferred PRO visuals in which a “good” score trended upwards. The majority of participants agreed that stoplight-colored graphs with corresponding color descriptions were the easiest to interpret. A majority of the youth and all crime victim specialists believed visual displays of anger, social, and emotional scores would be most helpful to see in a graphical format during their time in the program. Further investigation is needed to determine how PRO visuals can be integrated into the current care plan in a violence intervention program.

## Materials and methods

### Participants/demographics (age, gender)

Children age 7–18 years who were active participants in Project Ujima programming and their crime victim specialists were eligible for inclusion. Eligible youth were direct victims of violence, next of kin of homicide victims, or had a family member who was a victim of violence. Only those Project Ujima participants who received regular programmatic evaluations that included PROs were included in this study; this ensured participant familiarity with both the process and questions asked in the PRO questionnaires. Youth with no previous exposure to PRO measures were excluded from the study. Targeting youth and families with prior exposure to these measures diminished the burden on participants by decreasing the time to complete the study and the background knowledge needed to participate.

### Setting

Project Ujima hosts an 8-week summer camp for youth enrolled in programming. Participants were recruited in this setting.

### Measures

The National Institute of Health’s Patient-Reported Outcomes Measurement Information System (PROMIS®) is a patient-reported outcome Health-related Quality of Life (HRQOL) tool with validated pediatric domains (Cella et al. 2007). The PedsQL™ is a multidimensional self-report HRQOL measure, with good reliability, validity, and responsiveness, developed and validated for use in children (Brandow et al. 2009; Mistry et al. 2007; Stevens and Gorelick 2001; Stevens et al. 2011; Vivier et al. 1994; Varni et al. 2003). The questionnaire includes items in the physical, emotional, social, and school functioning domains (Stevens and Gorelick 2001; Vivier et al. 1994; Varni et al. 2003, 2001). Youth participants in Project Ujima complete the generic version of the PedsQL™ adapted to the PROMIS® web interface with permission from the proprietors of PedsQL™. This tool is a reliable and precise measurement system of HRQOL including social well-being and mental health status (Cella et al. 2007).

### Procedure

Youth and crime victim specialists were recruited for this qualitative study via convenience sampling at Project Ujima’s summer camp. Patients and crime victim specialists who consented to participate were assigned anonymous identification numbers. Program participants gave assent, and their parents gave their consent. The research procedure was approved by the Children’s Wisconsin Institutional Review Board. Research personnel administered qualitative, focused interviews with patients 7 years of age and older to match the age of PRO self-report data in our prior studies.

Each structured interview began with a brief review of the PRO measure questions for participants who had previously taken part in reporting PROs. Study personnel provided the participants with a list of currently collected PROs and assessed which domains and scores the youth preferred to see as part of their participation during their time in the violence intervention program. If youth did not understand a term, it was defined for them. A series of mock visuals (not actual patient data) (Fig. 1) displaying the data in a variety of formats was shared with the participants. Participants were asked which formats were preferred and easiest to understand. Participants were asked several additional open-ended questions to probe for comments or suggestions for PRO presentation. The corresponding questions are displayed (Fig. 1). Structured interview content was recorded, and descriptive statistics were used for all quantitative analyses. Participants were reimbursed for their time.

## Data Availability

All data generated or analyzed during this study are included in this published article.

## Abbreviations

HRQOL: Health-related Quality of Life
PROMIS®: The National Institute of Health’s Patient-Reported Outcomes Measurement Information System
PedsQL™: Pediatric Quality of Life Inventory
PROs: Patient-reported outcomes
